# Inflammation-induced Gro1 triggers senescence in neuronal progenitors: effects of estradiol

**DOI:** 10.1186/s12974-018-1298-y

**Published:** 2018-09-11

**Authors:** Svetlana Zonis, Joshua J. Breunig, Adam Mamelak, Kolja Wawrowsky, Catherine Bresee, Nadiya Ginzburg, Vera Chesnokova

**Affiliations:** 1 Pituitary Center, Department of Medicine, Cedars-Sinai Medical Center, 8700 Beverly Blvd., Los Angeles, CA 90048 USA; 2 Department of Neurosurgery, Cedars-Sinai Medical Center, 8700 Beverly Blvd., Los Angeles, CA 90048 USA; 3 Department of Biomedical Science, Cedars-Sinai Medical Center, 8700 Beverly Blvd., Los Angeles, CA 90048 USA; 4 Biostatistics and Bioinformatics Core, Cedars-Sinai Medical Center, 8700 Beverly Blvd., Los Angeles, CA 90048 USA

**Keywords:** Chemokines, Gro1, Hippocampus, Neuronal progenitor cells, Senescence, LPS-induced inflammation, Sex dimorphism

## Abstract

**Background:**

Inflammation has been proposed to contribute to the decline in adult hippocampal neurogenesis. Proinflammatory cytokines activate transcription of chemokine growth-regulated oncogene α (Gro1) in human and murine hippocampal neuronal progenitor cells (NPC). The goal of this study was to investigate the effects of Gro1 on hippocampal neurogenesis in the presence of inflammation.

**Methods:**

Human hippocampal NPC were transfected with lentivirus expressing Gro1, and murine NPC and hippocampal neuronal HT-22 cells were treated with Gro1 protein. A plasmid expressing mGro1 was electroporated in the hippocampus of newborn mice that were sacrificed 10 days later. Adult male and female mice were injected with lipopolysaccharide (LPS; 1 mg/kg, i.p in five daily injections) or normal saline. Adult male mice were implanted with pellets releasing 17-β estradiol (E2; 2.5 mg/pellet, 41.666 μg/day release) or placebo for 6 weeks and challenged with LPS or normal saline as above. In both experiments, mice were sacrificed 3 h after the last injection. Hippocampal markers of neurogenesis were assessed in vitro and in vivo by Western blot, real-time PCR, and immunohisto/cytochemistry.

**Results:**

Gro1 induced premature senescence in NPC and HT-22 cells, activating senescence-associated β-galactosidase and the cell cycle inhibitor p16 and suppressing neuroblast proliferation and expression of doublecortin (DCX) and neuron-specific class III beta-tubulin (Tuj-1), both neuroblast markers, while promoting proliferation of neural glial antigen 2 (Ng2)-positive oligodendrocytes. Gro1 overexpression in the hippocampus of newborn mice resulted in decreased neuroblast development, as evidenced by decreased DCX expression and increased expression of platelet-derived growth factor α receptor (PDGFαR), a marker of oligodendrocyte precursors. In adult mice, Gro1 was induced in response to LPS treatment in male but not in female hippocampus, with a subsequent decrease in neurogenesis and activation of oligodendrocyte progenitors. No changes in neurogenesis were observed in females. Treatment with E2 blunted LPS-induced Gro1 in the male hippocampus.

**Conclusions:**

Inflammation-induced Gro1 triggers neuroblast senescence, thus suppressing new neuron development in the hippocampus. Sex-dependent differences in Gro1 response are attributed to estradiol, which blunts these changes, protecting the female hippocampus from the deleterious effects of inflammation-induced Gro1 on neurogenesis.

## Background

The subgranular zone (SGZ) of the hippocampus maintains the capacity for neurogenesis throughout life [1, 2]. Radial glia-like quiescent neural stem cells express nestin or/and sex-determining region Y-box 2 (Sox2) as well as glial fibrillary acidic protein (GFAP), which, in turn, give rise to proliferating amplifying neuronal progenitors [3, 4]. In a process of differentiation, cells committed to a neuronal lineage lose these markers and acquire markers associated with immature neurons or neuroblasts, such as doublecortin (DCX), neuron-specific class III β-tubulin (recognized by Tuj-1 antibody), and polysialylated-neural cell adhesion molecule (PSA-NCAM). In vitro, most hippocampal progenitors become neurons; therefore, hippocampal neural stem cells are typically referred to as “neuronal progenitor cells” (NPC) [2, 5]. However, in vivo, ~ 25% of NPC differentiate into glial cells, comprising astrocytes and oligodendrocytes, and express cell-specific markers [4]. Astrocytes express the glial fibrillary acidic protein (GFAP) and S100 calcium-binding protein B (S100β) [4]. Oligodendrocyte precursors express platelet-derived growth factor α receptor (PDGFαR), neural glial antigen 2 (Ng2), and oligodendrocyte transcription factor 1 (Olig1) and Olig2, while mature oligodendrocytes lose these markers and begin to express O4 [6]. Over time, these newly added neurons incorporate into the functional hippocampal circuitry.

In animal models, abnormal hippocampal neurogenesis has been attributed to cognitive impairment, spatial memory, and learning deficits [7], and its potential role in depression [8, 9] has been widely discussed. Little is known about the role of neurogenesis in the normal adult human hippocampus, despite studies showing the presence of hippocampal neurogenesis in both human and primate adult brain [10–13]. Low proliferation capacity of human hippocampal NPC isolated from surgically removed specimens correlates with memory dysfunction in these patients [14], and antidepressant treatment significantly increases NPC in both murine and human hippocampus [8, 15, 16], suggesting that changes in NPC can have functional consequences in adult humans as well.

It has been shown that inflammation contributes to the decline in adult hippocampal neurogenesis [17–19]. Microglial cells, which are resident macrophages in the central nervous system (CNS), respond to signals from the peripheral immune system or to local insults, inducing neuroinflammation and releasing proinflammatory cytokines [20]. Thus, mice treated with bacterial endotoxin lipopolysaccharide (LPS) mimicking acute and intensive systemic inflammation demonstrate upregulation of multiple proinflammatory cytokines that can negatively affect hippocampal neurogenesis [18, 21–23].

Chemokines are induced in astrocytes and activated microglia in response to injury [24, 25], attract immune cells to sites of tissue damage, and enhance the inflammatory response by inducing the release of inflammatory cytokines and chemokines by neutrophils [26].

Chemokine growth-regulated oncogene α Gro1, also known as C-X-C motif ligand (CXCL) 1 or keratinocyte-derived chemokine (KC), signals through G protein-coupled receptor CXC receptor 2 (CXCR2); the human ortholog of Gro1 is interleukin (IL)-8, or CXCL8. (Both human CXCL8 and murine CXCL1 protein are here referred to as Gro1.) Gro1 is expressed in neurons and endothelial cells during status epilepticus in rats [27] and in mice after inflammatory stimuli in both endothelial cells and astrocytes [28–32]. In humans, Gro1 is induced in the brains of sepsis patients [33]. In vitro, cytokines interleukin (IL)-1β and tumor necrosis factor α (TNFα) induce Gro1 in murine astrocytes [34] and neuronal precursors [35]*.*

Gro1 transcription is regulated by nuclear factor kappa-light-chain-enhancer of activated B cells (NFκB) transcription factor, which, in turn, is activated by proinflammatory cytokines such as IL-1β, IL-6, and TNFα. NFκB-dependent Gro1 induction has been observed in multiple cell types, including neurons [36], pancreatic β cells [37], and a melanoma cell line [38].

Chemokines and their receptors are also expressed in the normal brain that is free of inflammation [39]. CXCR2 is constitutively expressed in NPC [40], and Gro1 and CXCR2 are involved in spatial and temporal regulation of oligodendrocyte proliferation in the spinal cord [41, 42] and promote oligodendrocyte maturation of neuronal stem cells [35]. Of note, Gro1 acts synergistically with PDGF expressed in astrocytes and neurons [41], stimulating proliferation of PDGFαR-positive oligodendrocyte progenitors and arresting their migration [43, 44].

Very little is known about the role of Gro1 in hippocampal neurogenesis. Here, we describe the effects of Gro1 on adult hippocampal human and murine NPC and on the HT-22 murine hippocampal neuronal cell line. We show that Gro1 activated the senescence pathway, as evidenced by induction of senescence-associated β-galactosidase (SA-β gal) and the cell cycle suppressing protein p16, and was associated with decreased expression of Ki67, a marker of proliferation. High Gro1 negatively affected neuronal lineage, decreasing proliferation of neuroblasts positive for Tuj-1 and DCX. Similar results were obtained in vivo, where electroporation of plasmid expressing Gro1 in the hippocampus of newborn mice led to decreased DCX expression, while PDGFαR was elevated.

We also show that Gro1 response to systemic inflammation is sex-dependent. Intraperitoneal injection of lipopolysaccharide (LPS) induced Gro1 expression in the male hippocampus, but the response was blunted in females, and treatment with 17-β estradiol (E2) reduced LPS-triggered Gro1 expression in male mice.

These findings outline new mechanisms underlying aberrant hippocampal neurogenesis and suggest a previously unknown sex-specific influence of Gro1 on neurogenesis during inflammation. These findings may be linked to age- and sex-specific differences in the incidence and symptoms of neuropsychiatric and neurodegenerative disorders.

## Methods

### Human samples

Human NPC were obtained using surgical hippocampal specimens of patients with mesial temporal lobe epilepsy undergoing partial removal of the hippocampus for an attempted surgical cure (Table 1).

**Table 1:** Patient characteristics

Patient #	Gender	Age, years	Surgical site	Seizure location method	Time from electrode insertion (weeks)	Age of seizure onset, years	Pathology
#1	Male	45	Right hippocampus	Depth electrodes	12	13	Hippocampal gliosis
#2	Male	44	Left hippocampus	Depth electrodes	10	22	Normal hippocampus
#3	Female	44	Right hippocampus	Depth electrodes	10	7	Hippocampal sclerosis and gliosis

### Experimental animals and treatments

#### In vivo electroporation

mGro1 plasmid was mixed with episomal pCagg-hyPbase and a transposable ubiquitin C promoter-driven EGFP reporter plasmid at a 1:0.7:1 ratio. This mix was delivered by injection through pulled glass capillary pipette to P0-P2 C57Bl/6 mice. Pups were then electroporated with electrodes positioned to target the right dorsal hippocampus. Employing Signagel, platinum Tweezertrodes were used for electroporation with 3–5 pulses of 115–135 V (50 ms; separated by 950 ms) generated with the ECM 830 BTX Electroporator (Harvard Apparatus) [45]. Control mice were electroporated with plasmid lacking Gro1. Animals were killed on day 10 after the procedure, the brains were immediately dissected in phosphate-buffered saline (PBS), and electroporated left halves of the brain were fixed in 4% PFA for at least 6 h at 4 °C. On the following day, the brains were embedded in 4% LMP agarose and sectioned at 70 or 250 μm thickness on a Leica VT1200S vibratome, and EGFP expression was analyzed with confocal Leica Sp5-X microscope. The whole hippocampi were isolated from the electroporated halves of the brain for further analysis.

#### LPS administration

Two-month-old C57Bl/6 male and female mice (Jackson Laboratory) were injected with LPS (1 mg/kg, in 200 μL of normal saline [NS], i.p.; Sigma-Aldridge) once a day for 5 days. Control mice received NS. Mice were killed 3 h after the last injection.

#### E2 pellets and LPS administration

Two-month-old C57Bl/6 male mice were surgically implanted under isoflurane anesthesia with E2 (2.5 mg/pellet, 41.666 μg/day release; Innovative Research of America, Sarasota, FL) or placebo pellets for 7 weeks. Five days before the mice were sacrificed, LPS or NS was injected as described above. Mice were killed 3 h after the last injection. There were thus four experimental groups: Placebo/NS, Placebo/LPS, E2/ NS, and E2/LPS.

### Adult NPC cultures and treatments

Human NPC was isolated from three individual surgical specimens (Table 1). All patients had electrodes (1.5 mm diameter) implanted in the region of the seizure focus prior to surgical resection, which were removed on average 10 weeks before surgery. The hippocampus was resected, and a piece of hippocampal tissue approximately 2 cm × 1 cm was placed into 30 mL of DMEM media (Corning-Cellgro cat#10-017-CV) with antibiotic-antimycotic (Gemini Bio-Products cat#400-101). Tissue was processed within an hour after dissection. Tissue was dissociated using Papain Dissociation System (Worthington Biochemicals). Cells were isolated according to published protocols [14, 46–48]. Human NPC were then cultured using NeuroCult NS-A Basal Medium (human, cat# 05750; StemCell Technologies) supplemented with NeuroCult NS-A Proliferation Supplement (human, cat #05753; StemCell Technologies) as well as 2 mM l-glutamine, 100 U/mL penicillin, 100 μg/mL streptomycin, 10 ng/mL h bFGF, and 20 ng/mL hEGF.

Murine NPC cultures were prepared according to published protocols [2, 15, 49, 50]. Murine NPC were isolated from ten pooled hippocampi of 2-month-old C57Bl/6 mice. NPC were cultured in NeuroCult NSC Basal Medium (mouse, cat#05700) with Proliferation Supplement (mouse, cat # 057012 mM), l-glutamine, 100 U/mL penicillin, 100 μg/mL streptomycin, 10 ng/mL m bFGF, and 20 ng/mL mEGF in Costar 6-Well Plate with Ultra-Low Attachment Surface (Corning, cat# 3471).

For both human and murine cultures, after 2 weeks in proliferation conditions, neurospheres were collected, cells dispersed by pipetting, and plated in plates pretreated with ECL cell Attachment Matrix (Upstate, 5–10 μg/cm^2^) at a density of 2.5–5 × 10^5^ per well in 6-well plates or 2–5 × 10^4^ per well in 24-well plates (Basal NeuroCult NSC media with Differentiation Supplement, StemCell Technologies, human cat# 05754, and mouse cat# 05703). Cells were placed in differentiating media and allowed to attach for 4–6 h and then treated. Both human and murine NPC were differentiated in the presence of 10 ng/mL IL-1β (Millipore, hIL-1β cat# IL038, mIL-1β cat# IL014) or 50 ng/mL IL-6 (Millipore, hIL-6 cat#IL006, mIL-6 cat# IL017) for 10 days. In some experiments, murine NPC was treated with mGro1 protein (Origen, cat# TP 723259) for 72 h. We used the dose of 80 ng/mL appeared to be the most effective in HT-22 cells (Fig. 7).

### Constructs and transfections

Lentiviral particles expressing human Gro1 (EF1-luc2-Gro1-Ubic) and control lentiviral particles were generated at the Cedars-Sinai Virus Core facility. Lentiviral particles expressing shmGro1 or shScr RNAi were purchased (Santa Cruz Biotechnologies).

For lentiviral transduction, cells growing in proliferating conditions were collected, plated into 6-well plates at a density of 5 × 10^5^ per well, placed in differentiating media, and allowed to attach to plastic for 4–6 h before 20 multiplicity of infection (MOI) of virus was added with 3 μg/mL polybrene (Santa Cruz Biotechnologies, cat# sc-134220). After 24 h, cells were washed and fresh media was added. Cells were collected 72 h after transduction.

### Cells

HT-22 (Millipore, cat# SCC129) is an immortalized mouse hippocampal cell line subcloned from the HT-4 cell line [51]. The parental HT-4 cell line was derived from the immortalization of mouse neuronal tissues with a temperature sensitive SV40 T-antigen [52]. HT-22 cells were cultured and propagated in DMEM with 10% FBS, 2 mM l-glutamine, 100 U/mL penicillin, 100 μg/mL streptomycin, and 20 ng/mL mEGF. Cells were treated with murine Gro1 and harvested after 72 h.

### Quantitative real-time polymerase chain reaction

Total RNA was isolated from the hippocampi with RNeasy Lipid Tissue Mini Kit (Qiagen, cat# 74804). cDNA was synthesized from 0.5 to 1 μg of purified RNA by iScript Reverse Transcription Supermix (Bio-Rad, cat# 1708841) according to the manufacturer’s instructions. Quantitative PCR was performed in 20 μL reaction using IQ SYBR Green Master Mix and CFX96 Real-Time System standard protocol (Bio-Rad Laboratories, Hercules, CA). Specific validated primers for murine DCX, Ng2, Gro1, and glyceraldehyde-3-phosphate dehydrogenase (GAPDH), as well as human Gro1, fibroblast growth factor 2 (FGF2), and glial cell-derived neurotrophic factor (GDNF), were purchased (SuperArray, Qiagen, Germantown, MD). Triplicate PCR reactions yielded threshold cycle (Ct) average, with a coefficient of variance of < 0.05%, which were used to determine ΔCt values [ΔCt = Ct of the target gene minus Ct of the housekeeping GAPDH gene]. A comparative threshold cycle (C_T_) method was used for relative gene expression quantification. All experiments included template-free (water) and reverse transcriptase-minus controls to ensure no contamination. Relative quantities of mRNA in experimental samples were determined, normalized to GAPDH, and expressed in arbitrary units as fold difference from control (control was taken as 1).

### Protein isolation and Western blot analysis

NPC growing in culture were collected in Trizol reagent (Thermo Fisher Scientific, Waltham, MA) and proteins isolated according to the protocol (Molecular Research Center) using radioimmunoprecipitation assay (RIPA) buffer (Cell Signaling, cat#9806) with Protease Inhibitor Cocktail (Sigma, cat# P8340). Western blot analysis was performed as described [22]. Thirty to fifty micrograms of protein lysate was resolved by SDS-PAGE and electroblotted onto polyvinylidene difluoride (PVDF) membrane (EMD Millipore, Billerica, MA). The membrane was blocked by 5% non-fat dry milk in Tris-buffered saline (TBST; 50 mM Tris-HCl, pH 7.6, 150 mM NaCl, 0.05% Tween 20) and incubated overnight with primary antibodies at 4 °C, followed by incubation with corresponding secondary antibodies (Sigma-Aldrich, St. Louis, MO) for 2 h at room temperature. Immunoreactive bands were detected using Bio-Rad Molecular Imager® ChemiDoc™ XRS and Image Lab™ Software (BioRad Laboratories). The following antibodies were used: Gro1 (Novus cat# NBP1-51188), SA-β-gal (LSBio cat# LS-B10989), DCX (Santa Cruz Biotechnologies, cat# sc-271390), Tuj-1 (Abcam,cat# ab182-07), Ng2 (Millipore, cat# AB5320), total p65 (Santa Cruz Biotechnologies, cat# sc-8008), phospho-p65 (Ser536; Cell Signaling, cat# 3033), murine Gro1 (R&D, cat# AF-453), GFAP (Millipore, cat# mAB3402), cleaved caspase 3 (Cell Signaling, cat# 9664), Ki67 (Abcam, cat# ab15580), p16 (Santa Cruz Biotechnologies, cat# sc-1661), ionized calcium-binding adapter molecule 1 (Iba1; Abcam, cat# ab107159), PDGFαR (LSBio, cat# LS-B6056), and GAPDH (Cell Signaling, cat #5174).

### Immunohistochemistry and immunocytochemistry

To detect Gro1 in SGZ of the hippocampus, the right half of the brain from three randomly selected mice was fixed and sections from 0.36 to 0.6 mm lateral to the midline [53] were cut. Five randomly selected slides from each mouse were analyzed. Paraffin-embedded brain sagittal sections (5 μm) were double-labeled with primary antibodies conjugated with Alexa 488 or Alexa 568 fluorescent dyes (1:400, Thermo Fisher). The following primary antibodies were used: mouse Gro1 (RnD, cat# AF-453), Sox2 (Millipore, cat#AB56030), DCX (Abcam, cat# ab18723), and GFAP (Millipore, cat# MAB3402 or Abcam, cat# ab7260). Nuclear DNA was stained with DAPI (Sigma, cat#D9542), and the stained sections were covered with ProLong Gold (Thermo Fisher, cat#P36935). Antigen retrieval was performed on paraffin-embedded tissues in Target Retrieval Solution (Dako, cat# S1699).

Neurospheres growing in proliferation media for 2 weeks were collected, dissociated by pipetting with a micropipette, and plated on coverslips pretreated with ECL matrix in 24-well plates in differentiation media. Cells were allowed to attach to coverslips for 4–6 h and then treated. Areas with the highest cell density were imaged with Leica × 20 Plan-Apo lens on a Leica Sp5-X confocal microscope. The number of double positive and total cells was counted in 5–15 fields in two independent experiments (total number of cells analyzed was between 500 and 3000 depending on the experiment). The following primary antibodies were used: Tuj-1 (Stem Cell Technology, cat# 01409 or Abcam, cat# ab182-07), Ng2 (Millipore, cat# AB5320), GFAP (Millipore, cat# MAB3402 or Abcam, cat# ab7260), Ki67 (Abcam, cat#ab15580), DCX (Abcam cat#ab18723), and p16 (Santa Cruz cat# sc-1207 or sc-1661).

### SA-β-galactosidase activity

SA-β-galactosidase enzymatic activity was assayed in vitro using a β-gal staining kit (Senescence Cell Staining Kit, Sigma-Aldrich) according to the manual. Briefly, 10,000 cells were plated in 12-well plates, treated for the indicated times, washed with PBS (pH 6.0), fixed, and stained with 5-bromo-4-chloro-3-indolyl-h-d-galactopyranoside (X-Gal) overnight at 37 °C. Only senescent cells stain at pH 6.0. SA-β-gal positivity was assessed in 6-well plates in triplicate, with 1000 cells per field counted in three fields per well.

### Statistical analysis

Differences in protein and mRNA levels were assessed by one or two-way ANOVA followed by Tukey’s test to correct for post-hoc multiple testing. The number of cells positive for Ki67, Ng2, Tuj-1, p16, or GFAP across the groups was assessed with a two-tailed *t* test. For all testing, data were log transformed prior to analysis where data were not normally distributed. Results were inspected to confirm fit. Differences were considered statistically significant at the two-tailed *p* value of < 0.05.

## Results

### Proinflammatory cytokines induce Gro1 in human and murine hippocampal NPC

**Fig. 1:**
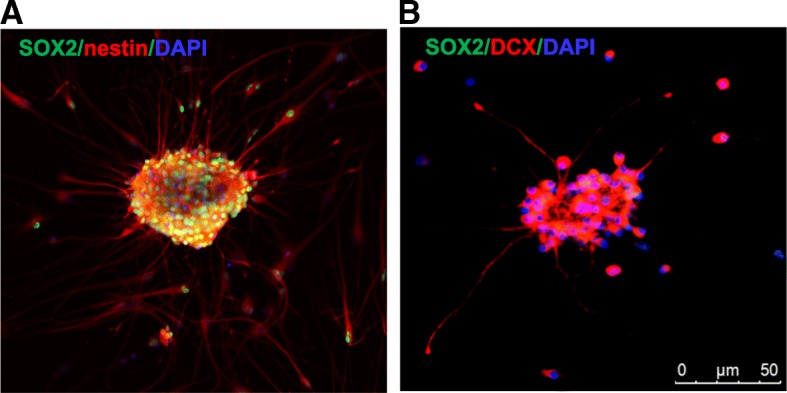
Confocal image of human NPC in culture. **a** Proliferative conditions. Cells express both SOX2 and nestin, markers of stem/early progenitor cells. **b** Differentiating conditions. After 1 week, NPC begin to lose stem cell marker Sox2 and to acquire neuroblast marker DCX

Human NPC were isolated individually from the hippocampal tissue derived from surgical samples of three patients with mesial temporal lobe epilepsy (Table 1). NPC formed spheres, which were cultured under proliferative conditions for 2 weeks. Cells were initially shown to express SOX2/nestin, markers of early neuronal progenitors. Cells were dispersed and plated under differentiating conditions for 1 week when they began to lose SOX2 and to acquire DCX, a marker of neuroblasts (Fig. 1). This confirmed progenitor properties of the NPC.

**Fig. 2:**
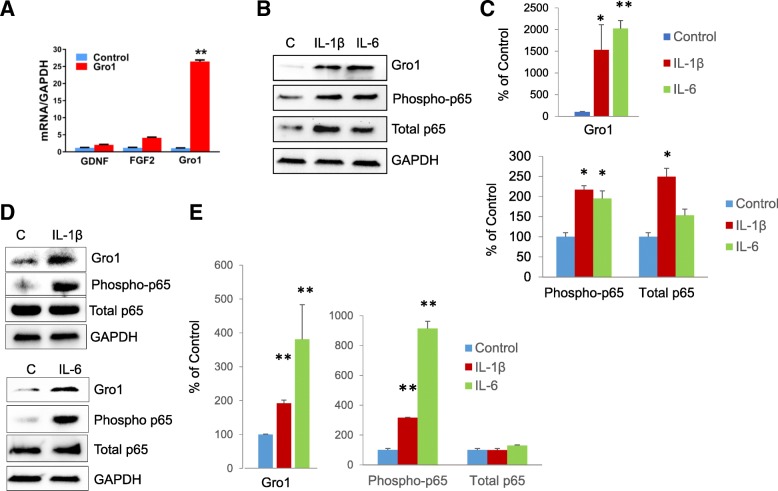
Proinflammatory cytokines induce Gro1 expression in human and murine hippocampal NPC. **a** mRNA levels of growth factors in hNPC treated with IL-1β (10 ng/mL) for 10 days. Data are shown as mean ± SEM of three independent experiments. All samples from three experiments were run together in triplicate and normalized against GAPDH. Results are expressed in fold change vs untreated control taken as 1; **p < 0.01. **b** Western blot analysis of Gro1 and NFκB subunits in hNPC treated with IL 1β (10 ng/mL) or IL 6 (50 ng/mL) for 10 days. C, untreated control. Three independent experiments were performed, and representative blots are shown. **c** Intensities of protein bands were quantified from three individual experiments, normalized to GAPDH, and presented as percent of control (untreated cells); *p < 0.05, **p < 0.01 vs control in post-hoc pair-wise *t* test. **d** Western blot analysis of Gro1 in mNPC treated with IL-1β (10 ng/mL) or IL-6 (50 ng/mL) for 5 days. C, untreated control. Three independent experiments were performed, and representative blots are shown. **e** Intensities of protein bands were quantified from three individual experiments, normalized to GAPDH, and presented as percent of control (untreated cells); *p < 0.05, **p < 0.01 vs control in post-hoc pair-wise *t* test

We first examined how the exposure of NPC to IL-1β affects the expression of growth factors. Differentiating cells were treated with IL-1β for 10 days. Real-time PCR revealed marked induction (26 ± 3-fold) of Gro1 mRNA levels in IL-1β-treated NPC compared to untreated controls (*t*(4) = 53.9, *p* < 0.0001), while growth factors for neuronal progenitors, including GDNF and FGF2, were not significantly induced (Fig. 2a).

Exposure of differentiating human NPC to IL-1β or IL-6 for 10 days also resulted in marked induction of Gro1 protein levels (*F*2,4 = 106.94, *p* = 0.0003 vs untreated cells, Fig. 2b,c). It is possible that Gro1 can be activated by cytokine-induced NFκB, which has been shown to increase Gro1 transcription [37, 54]. Phosphorylation of p65 (RELA) subunits is required for transcriptional NFκB activation [36]; in human NPC, we found upregulated expression of both total (*F*2,4 = 330.26, *p* < 0.0001) and phospho-p65 (*F*2,4 = 108.81, *p* = 0.0001) subunits after treatment with IL-6 and with IL-1β (Fig. 2b, c).

We repeated these experiments with murine NPC isolated from the hippocampus of 2-month-old mice and found significant upregulation of phospho-p65 (*F*2,7 = 74.73, *p* < 0.001) followed by induction of Gro1 after 10 days of exposure to IL-1β or IL-6 (*F*2,7 = 34.3, *p* = 0.0002) (Fig. 2d, e).

### Gro1 suppresses new neuron development in human and murine NPC

We next examined how upregulated Gro1 affects neurogenesis. Human NPC were infected with hGro1-expressing lentivirus and cultured for 72 h under differentiating conditions.

**Fig. 3:**
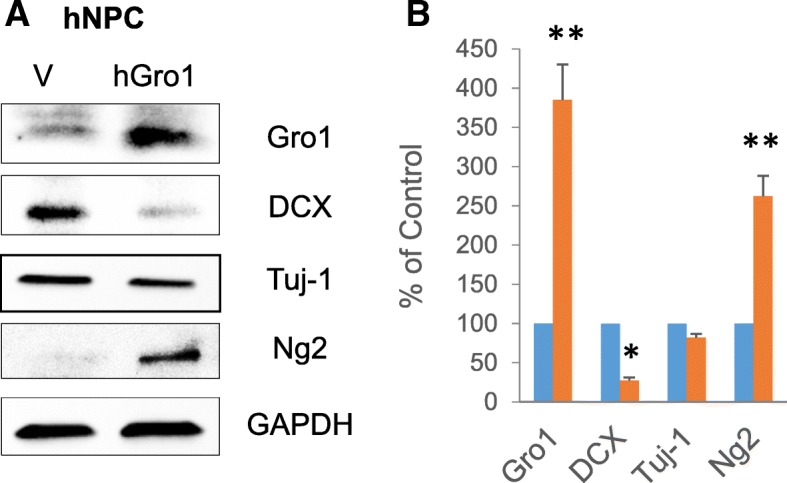
Gro1 suppresses new neuron development in human NPC. **a** Western blot analysis of neurogenesis markers. Human NPC (hNPC) were infected with lentivirus expressing human Gro1 (hGro1) or empty vector (V). Three independent experiments were performed, and representative blots are shown. **b** Intensities of protein bands were quantified from three individual experiments, normalized to GAPDH, and presented as percent of control (empty vector); *p < 0.05, **p < 0.01. vs control in post-hoc pair-wise *t* test

Gro1 overexpression significantly affected the neuronal markers (*F*1,2 = 65.39, *p* = 0.0149), resulting in decreased levels of neuroblast marker DCX (*F*1,2 = 78.47, *p* = 0.0125), while Ng2, a marker of oligodendrocyte progenitors, was upregulated (*F*1,2 = 476.61, *p* = 0.0023) (Fig. 3a, b).

### Gro1 induces senescence in neuroblasts

**Fig. 4:**
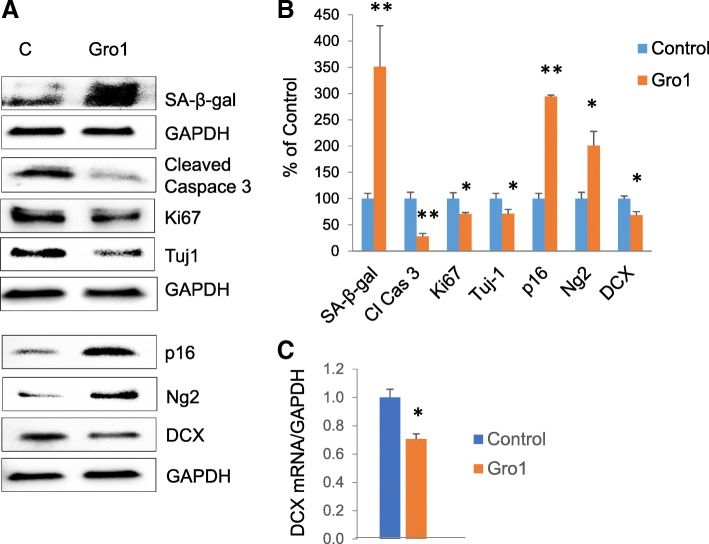
Gro1 induces senescence in neuroblasts. **a** Western blot analysis of markers of neurogenesis, senescence, apoptosis, and proliferation in murine NPC treated with mGro1 protein (80 ng/mL). Three independent experiments were performed, and representative blots are shown. C, untreated control. **b** Intensities of protein bands were quantified from three individual experiments, normalized to GAPDH, and presented as percent of control (untreated cells). **c** DCX mRNA levels in mNPC treated with Gro1. Data are shown as mean ± SEM of three independent experiments. All samples from three experiments were run together in triplicate and normalized against GAPDH. Results are expressed in fold change vs control (untreated cells) taken as 1. *p < 0.05, **p < 0.01 vs control in post-hoc pair-wise *t* test

We further assessed the mechanisms underlying Gro1-induced loss of neuroblasts. As Gro1 is a secreted protein, and neuronal progenitors express Gro1 receptor CXCR2 [40], we isolated NPC from murine hippocampus and treated them with mGro1 protein (80 ng/mL) for 72 h. Control cells were untreated. This dose was identified as the most effective in HT-22 cells, as described below. mGro1 treatment resulted in strong induction of SA-β-gal (*F*1,2 = 41.93, *p* = 0.0230), an ultimate marker of senescence [55, 56], as well as induced expression of the cyclin-dependent kinase inhibitor p16 (*F*1,2 = 33,776, *p* < 0.0001), indicating activation of the senescence pathway and proliferation arrest. Indeed, Ki67, a marker of proliferation, was downregulated in Gro1-treated cells (*F*1,2 = 48.42, *p* = 0.0200), as was the neuroblast marker DCX (*F*1,2 = 26.46, *p* = 0.0358) (Fig. 4a, b). Tuj-1, a marker of newly developing neurons, was also suppressed (*F*1,2 = 281.69, *p* = 0.035), while the oligodendrocyte progenitor marker Ng2 was induced (*F*1,2 = 55.30, *p* = 0.0176). As apoptosis is suppressed in senescent cells, we observed decreased expression of cleaved caspase 3 in NPC treated with Gro1 (*F*1,2 = 225.59, *p* = 0.0044) (Fig. 4a, b). To examine the effects of Gro1 on DCX transcription, we assessed DCX mRNA by real-time PCR and observed ~ 40% downregulation, confirming a decrease in neurogenesis after Gro1 treatment (*F*1,2 = 206.76, *p* = 0.048, Fig. 4c).

**Fig. 5:**
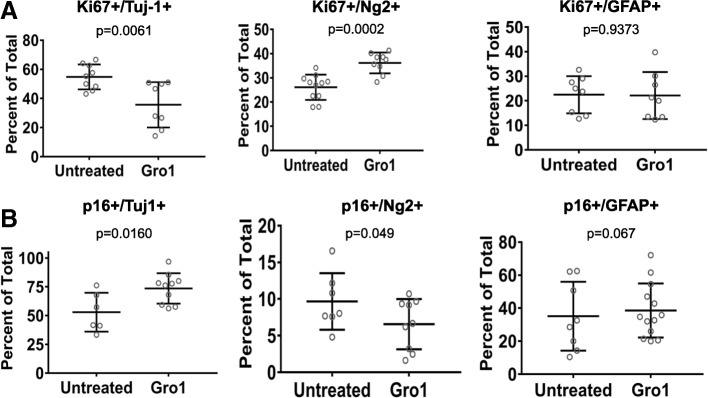
Gro1 suppresses proliferation of neuroblasts and increases proliferation of oligodendrocytes. Immunocytochemistry of mNPC treated with Gro1 (80 ng/mL) for 72 h or left untreated. **a** Cells were co-stained for Ki67- and Tuj-1, Ng2, or GFAP. The graphs depict the percentage of Ki67+ proliferating cells among Tuj-1+ neuroblasts, Ng2+ oligodendrocyte precursors, or GFAP+ astrocytes. **b** Cells were co-stained for p16, Tuj-1, Ng2, and GFAP. The graphs depict the percentage of p16+ non-proliferating cells among Tuj-1+ neuroblasts, Ng2+ oligodendrocyte precursors, or GFAP+ astrocytes. Counting was performed in triplicate (> 500 cells per sample). Data were tested across groups using *t* test or Wilcoxon rank-sum test. Data are presented as a mean ± SEM

**Fig. 6:**
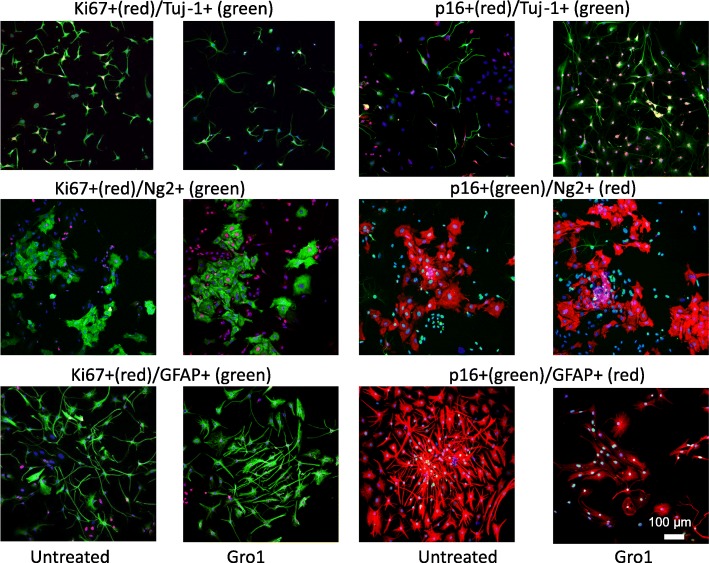
Gro1 suppresses proliferation of neuroblasts and increases proliferation of oligodendrocytes. Confocal image of murine NPC under differentiating conditions treated with Gro1. Cells were double-labeled with either Ki67 or p16, and Tuj-1, Ng2, and GFAP. Representative images are shown

To confirm cells undergoing senescence were neuroblasts, we performed immunocytochemistry studies to assess the number of proliferating cells expressing neuroblast and glial markers. Treatment of differentiating NPC with Gro1 for 72 h markedly decreased the percentage of Tuj-1+ neuroblasts positive for Ki67 (*t*(15) = − 3.9, *p* = 0.0061), while the number of Ng2+/Ki67+ oligodendrocyte progenitor cells was increased (*t*(18) = 4.64, *p* = 0.0002). By contrast, no changes were observed in GFAP+/Ki67+ cells between treated and untreated groups, indicating that the proliferation rate of astrocytes did not change (Figs. 5a and 6). Moreover, we found increased numbers of Tuj-1-positive neuroblasts expressing p16 (*t*(14) = 2.74, *p* = 0.0160) and decreased numbers of Ng2+/p16+ cells (*t*(18) = − 3.8, *p* = 0.049) (Figs. 5b and 6). These results show that the effects of Gro1 in NPC are cell type-specific, inducing proliferation arrest and likely senescence in neuroblasts while increasing oligodendrocyte progenitor proliferation.

### Gro1 induces senescence and arrest proliferation in murine hippocampal neurons

**Fig. 7:**
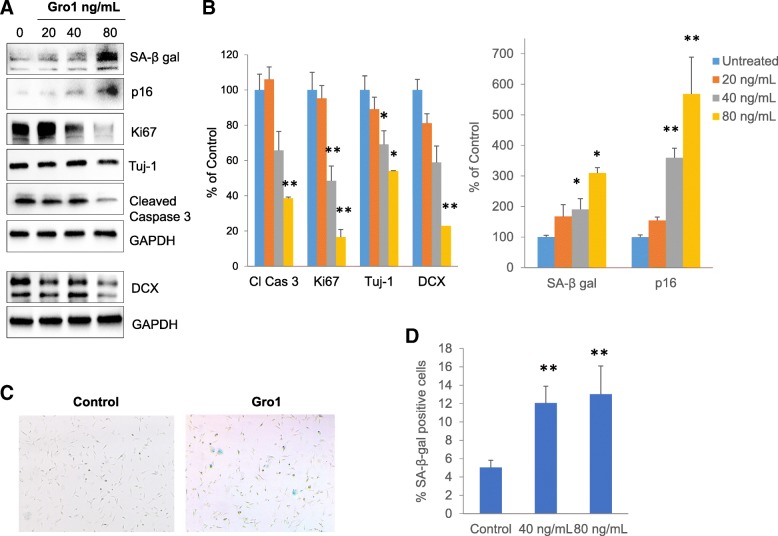
Gro1 induces senescence and arrests proliferation of murine hippocampal neurons. **a** Western blot analysis of markers of neurogenesis, senescence, apoptosis, and proliferation in HT-22 cells treated with mGro1. Three independent experiments were performed, and representative blots are shown. **b** Intensities of protein bands were quantified from three individual experiments, normalized to GAPDH, and presented as percent of control (untreated cells). **c** SA-β-gal enzymatic activity (blue) in HT-22 cells treated with 80 ng/mL Gro1. **d** Percent SA-β-gal positivity in HT-22 cells treated with Gro1 assessed in 6-well plates in triplicate, with 1000 cells/field counted in three fields/well. Control cells were untreated. *p < 0.05, **p < 0.01 vs control in post-hoc pair-wise *t* test

To further confirm that Gro1 acts to arrest neuronal cell proliferation, we tested the effects of Gro1 in the immortalized hippocampal neuronal cell line HT-22. These cells exhibit properties of newly developing neurons, as they lack the cholinergic and glutamate receptors seen in mature neurons [51, 52], yet still express DCX and Tuj-1, which are markers of immature neurons. Cells were treated with increasing doses of Gro1 or left untreated and analyzed after 72 h. Gro1 dose-dependently induced SA-β gal (*F*3,6 = 16.54, *p* = 0.0026) as well as p16 (*F*3,6 = 20.76, *p* = 0.0014) expression, while Ki67 expression was inversely downregulated (*F*3,6 = 29.38, *p* = 0.0006) and expression of cleaved caspase 3 was reduced (*F*3.6 = 15.59, *p* = 0.031); treatment with the 80 ng/mL dose of Gro1 also resulted in decreased expression of DCX (*F*3,9 = 15.81, *p* = 0.0006) and Tuj-1 (*F*3,6 = 16.25, *p* = 0.0028) (Fig. 7a, b). The number of SA-β-gal-positive senescent cells in response to Gro1 treatment was markedly increased (*F*2,15 = 21.16, *p* < 0.0001) (Fig. 7c, d), indicating that Gro1 induces senescence, decreases apoptosis, and arrests proliferation of neuronal cells, likely through p16 induction.

### Gro1 suppresses neurogenesis in the hippocampus of newborn mice

**Fig. 8:**
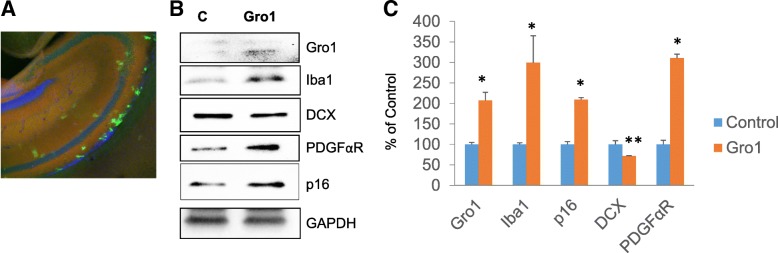
Gro1 suppresses hippocampal neurogenesis in vivo. **a** Confocal image of murine hippocampus 10 days after in vivo Gro1 electroporation. Green (EGFP), Gro1-expressing plasmid. **b** Representative Western blot analysis of hippocampal neurogenesis in mice electroporated with plasmid expressing mGro1 or control (C, empty vector). **c** Intensities of protein bands were quantified from three individual mice/group, normalized to GAPDH, and presented as percent of control (empty vector). *p < 0.05, **p < 0.01 vs control in post-hoc pair-wise *t* test

As a proof of concept, we electroporated a plasmid expressing mGro1 into the brain of 1-day-old C57Bl/6 mice; control mice were electroporated with a plasmid expressing empty vector. Ten days later, mice were sacrificed and the hippocampi isolated. Gro1 expression in the hippocampus was confirmed by visualizing EGFP reporter vector on confocal microscopy (Fig. 8a). Hippocampal Gro1 electroporation led to increased Gro1 (*F*1,2 = 337.05, *p* = 0.030) and Iba1 expression, a marker of activated microglia (*F*1,2 = 22.73, *p* = 0.0413). We also observed a marked decrease in DCX expression (*F*1,2 = 815.04, *p* = 0.0012), indicative of decreased neuroblast number and confirming our in vitro finding that Gro1 suppresses neurogenesis. PDGFαR, a marker of early oligodendrocyte precursors, was upregulated (*F*1,2 = 25.21, *p* = 0.0375) in agreement with the pro-proliferative effect of Gro1 on early-stage oligodendrocytes [44]. Of note, SA-β-gal was undetectable (not shown), but p16 was induced in the hippocampus of mice treated with Gro1 (*F*1,2 = 50.26, *p* = 0.0193) (Fig. 8b, c).

### Gro1 suppression results in decreased senescence and the induction of neuronal markers in murine NPC

**Fig. 9:**
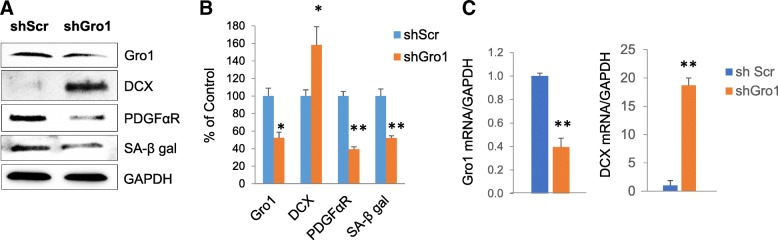
Gro1 suppression leads to increased expression of neuroblast marker DCX and decreased senescence. **a** Western blot analysis of mNPC infected with lentivirus expressing shGro1 or sh scramble (shScr) RNAi. **b** Intensities of protein bands were quantified from three individual experiments, normalized to GAPDH, and presented as percent of control (shScr). **c** Gro1 and DCX mRNA levels in mNPC infected with lentivirus expressing shGro1 or shScr RNAi. Data are shown as mean ± SEM of three independent experiments. All samples from three experiments were run in triplicate and normalized against GAPDH. *p < 0.05, **p < 0.01 vs control in post-hoc pair-wise *t* test

To examine the effects of Gro1 suppression on NPC differentiation, we infected murine NPC with lentivirus expressing shGro1 RNAi and analyzed cells 72 h later. Gro1 downregulation was confirmed by both Western blot (*F*1,3 = 17.48, *p* = 0.0249) and real-time PCR (*F*1,2 = 815.47, *p* = 0.0012) (Fig. 9a–c). Cells with suppressed Gr01 showed upregulated DCX protein (*F*1,4 = 13.96, *p* = 0.0202) and mRNA levels (*F*1,2 = 52,863, *p* < 0.0001) (Fig. 9a–c). SA-β gal was also markedly reduced (*F*1,2 = 159.75, *p* = 0062), likely resulting in new neuron development (9a–c). At the same time, PDGFαR expression was downregulated (*F*1,2 = 21.91, *p* = 0.0427) (Fig. 9a, b) in agreement with observed pro-proliferative effects of Gro1 on early oligodendrocyte progenitors [35]. Thus, Gro1 suppression results in increased neurogenesis and decreased oligodendrogenesis.

### Gro1 is expressed in early neuronal progenitors in the SGZ of the hippocampus

**Fig. 10:**
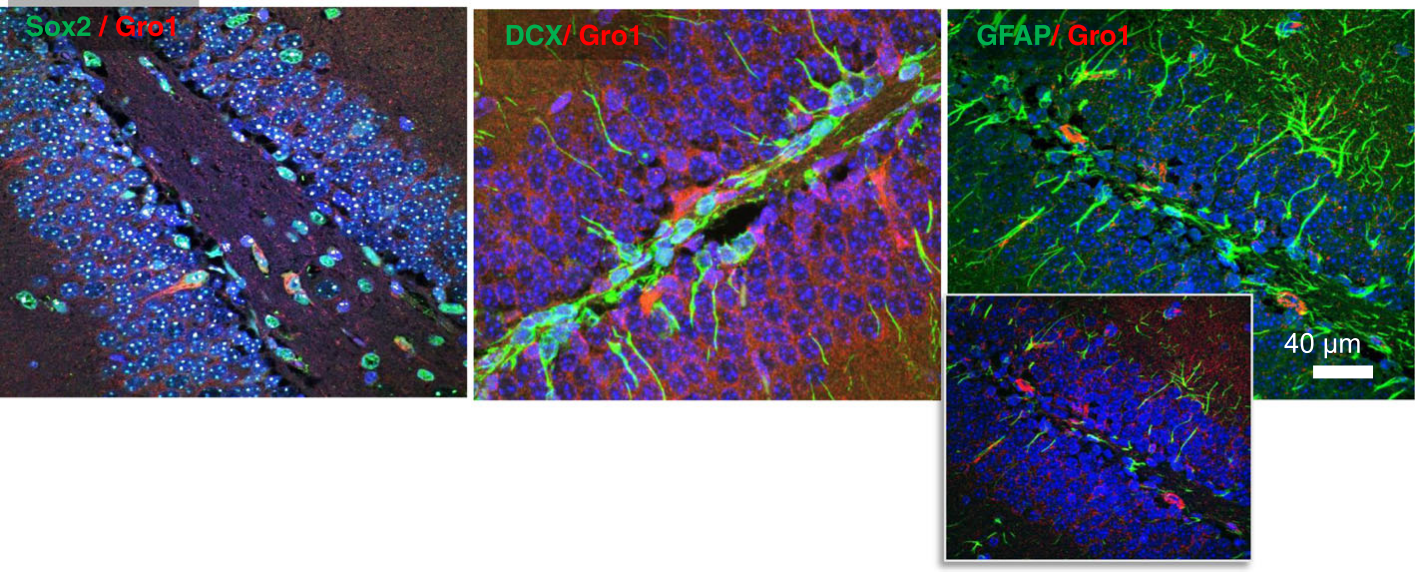
Gro1 is expressed in Sox2-positive early neuronal progenitors. Representative confocal images of the SGZ in the adult murine hippocampus. Gro1 (red) is co-localized with Sox2 (green; left), but not with DCX (green; center) or GFAP (green; right). Inset with a dimmed green channel for better visualization demonstrates lack of co-localization between GFAP and Gro1

We analyzed the expression of Gro1 at the site of neurogenesis in the SGZ of the dentate gyrus of the adult murine hippocampus. Gro1 was expressed in cells expressing SOX2, an early marker of neuronal progenitors, while no co-localization of Gro1 with DCX+ neuroblasts or GFAP+ astrocytes was observed (Fig. 10). These results suggest that Gro1 is expressed by and likely secreted from early progenitors, affecting SGZ cell differentiation.

### Gro1 is markedly induced in the male hippocampus and blunted in females during systemic inflammation

To test the effects of systemic inflammation on Gro1 expression in the hippocampus, 2-month-old male and female mice were injected with 1 mg/kg LPS i.p. once daily for 5 days. Control mice received NS. Mice were sacrificed 3 h after the last injection.

**Fig. 11:**
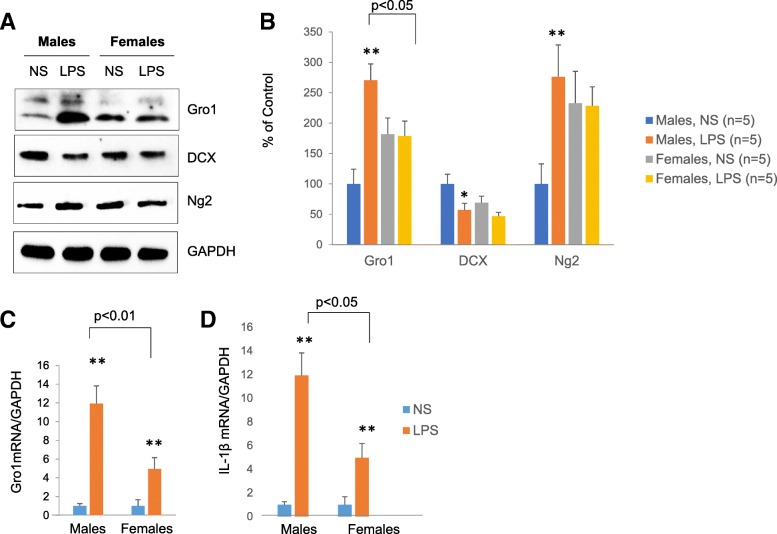
Gro1 is markedly induced in the male hippocampus and blunted in females in response to LPS. **a** Western blot analysis of the hippocampus of male and female mice treated with LPS (1 mg/kg i.p., once daily for 5 days) or normal saline (NS). Five mice per group were treated. Representative blots are shown. **b** Intensities of protein bands were quantified from five individual mice/group, normalized to GAPDH, and presented as percent of control (NS). *p < 0.05, **p < 0.01 vs control in a post-hoc pair-wise *t* test. **c** Gro1 mRNA and **d** IL-1β mRNA levels in the hippocampus of LPS or NS-treated mice. NS control is taken as 1. Data are shown as mean ± SEM. Samples from five mice/group were run in triplicate and normalized against GAPDH. Results are expressed in fold change vs control (NS) taken as 1. *p < 0.05, **p < 0.01 vs control in post-hoc pair-wise *t* test

Two-way ANOVA analysis revealed the significant interaction of sex and LPS treatments for expression of Gro1 (*F*1,22 = 6.31, *p* = 0.0198), DCX (*F*1,22 = 5.02, *p* = 0.0355), and Ng2 (*F*1,22 = 14.10, *p* = 0.0011). Compared to NS control, the male hippocampus after LPS treatment showed significantly upregulated Gro1 expression (*t*(22) = 4.65, *p* = 0.007) as well as decreased DCX (*t*(22) = − 5.54, *p* < 0.001) and increased Ng2 protein levels (*t*(22) = 6.84, *p* < .0001) (Fig. 11a–c). These data support our hypothesis that elevated Gro1 is associated with decreased neurogenesis. However, in females, LPS did not induce Gro1 expression, and no significant changes in DCX and Ng2 were observed compared to control females injected with NS (Fig. 11a–c).

Significant effects of sex (*F*1,14 = 4,6, *p* = 0.0488) and LPS treatment (*F*1,15 = 1020, *p* < 0.0001) were also seen in Gro1 mRNA response. Gro1 mRNA levels were increased in the female hippocampus in response to inflammation, albeit at a much lower rate than in male mice (*t*(15) = 22.4, *p* < 0.001) (Fig. 11c).

We also measured IL-1β expression in the hippocampus of males and females after LPS treatment and found significantly higher IL-1β mRNA levels in male than in female hippocampus (*t*(15) = 20.20, *p* < 0.001) (Fig. 11d). These results suggest a sexual dimorphism in neurogenesis in response to inflammation.

### E2 treatment suppresses LPS-induced Gro1

**Fig. 12:**
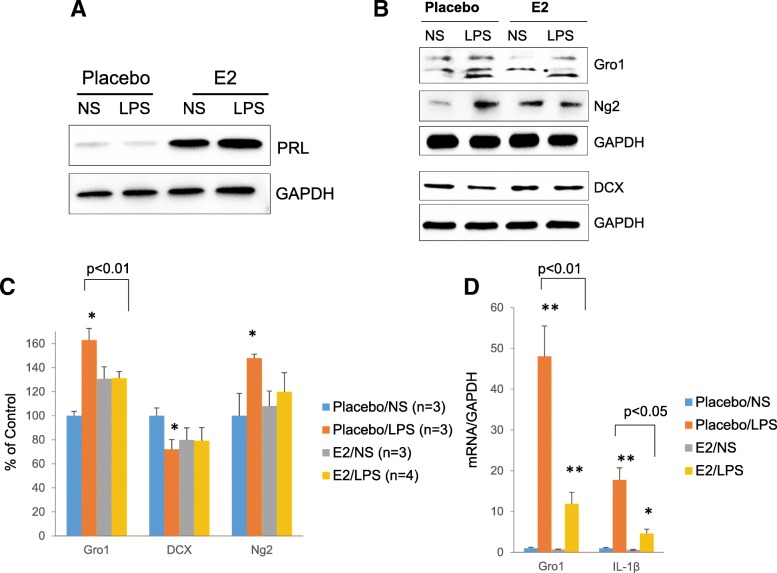
E2 treatment suppresses LPS-induced Gro1. **a** Western blot analysis of pituitary and **b** hippocampus of male mice treated with E2 or placebo pellets for 6 weeks and challenged with LPS or normal saline (NS) as in Fig. 11. Representative blots are shown. **c** Intensities of protein bands were quantified from three to four individual mice/group, normalized to GAPDH, and presented as percent of control (Placebo/NS). **d** Gro1 mRNA and IL-1β mRNA levels in the hippocampus. Data are shown as mean ± SEM. Samples from three to four mice/group were run in triplicate and normalized against GAPDH. Results are expressed in fold change vs control (Placebo+NS) taken as 1. *p < 0.05, **p < 0.01 vs control in post-hoc pair-wise *t* test

To test whether differential responses to inflammation are attributed to female sex hormones, 2-month-old male mice were implanted with pellets releasing E2 or placebo for 6 weeks. The concentration of circulating E2 released by the pellet is high for males but is half of the E2 level observed in pregnant females [57]. Mice were challenged with LPS or NS as described above and sacrificed. Mice were divided into four experimental groups: Placebo/ NS, Placebo/LPS, E2/NS, and E2/LPS. As E2 is a major regulator of pituitary prolactin [58], the efficacy of the E2 treatment was confirmed assessing prolactin protein levels in the pituitary of experimental mice (Fig. 12a). Analysis showed significant crossover interaction between E2 and LPS treatments for Gro1 (*F*1,16 = 5.57, *p* = 0.0313), DCX (*F*1,22 = 14.81, *p* = 0009), and Ng2 (*F*1,16 = 14.68, *p* = 0.0015). Compared to placebo-treated mice, E2-treated mice showed blunted hippocampal Gro1 response to LPS. In Placebo/LPS mice, Gro1 protein was induced ~ 60% relative to control (Placebo/NS) (*t*(16) = 4.79, *p* = 0.0010), while no induction was observed in E2/LPS mice (Fig. 12b, c).

Similarly, there was a significant interaction between E2 and LPS on LPS-induced Gro1 mRNA levels (*F*1,9 = 7.08, *p* = 0.0260). In the LPS group, Gro1 mRNA expression was lower in E2-treated mice compared to placebo-treated mice (*t*(9) = − 4.56, *p* < 0.0061) (Fig. 12d).

Of note, in Placebo/LPS mice, Gro1 induction was accompanied by decreased DCX (*t*(16) = 4.99, *p* = 0.0001) and increased Ng2 (*t*(16) = 4.99, *p* = 0.0007) protein levels compared to Placebo/NS controls, indicating a decreasing proliferation of neuroblasts and increasing proliferation of oligodendrocyte precursors. However, no differences in DCX or Ng2 were observed between E2/LPS- and E2/NS-treated mice (Fig. 12b, c). Thus, blunted Gro1 response to LPS in the female hippocampus is at least partially due to the suppressive effects of estradiol.

Similar to what was observed in females, levels of IL-1β mRNA were significantly lower in the hippocampus of E2/LPS male mice as compared to control Placebo/LPS mice (*t*(9) = − 4.0, *p* = 0.023) (Fig. 12d), suggesting that E2 may suppress LPS-triggered Gro1 activation by inhibiting IL-1β expression in hippocampus.

## Discussion

We show here that the chemokine Gro1 induced in response to inflammation triggers senescence and arrests development of new neurons in the hippocampus and that the magnitude of this response is sex-dependent.

In mouse brain, Gro1 is expressed in Sox2-positive neuronal progenitors, but not in DCX-positive neurons or astrocytes. In both human and murine NPC, and in the murine hippocampus, inflammatory stimuli induce Gro1 transcription and translation.

Our results indicate that Gro1 triggers premature senescence in newly developing neurons. Cellular senescence is a state in which the cell stays metabolically active but loses the ability to proliferate in response to growth factor. Cell cycle arrest usually occurs upon activation of cell cycle kinase inhibitors such as p21 or p16 [59]. Treatment with Gro1 resulted in marked induction of the senescence marker SA-β gal in hippocampal NPC, along with decreased Ki67 and induced p16 indicating decreased cell proliferation. DCX and Tuj-1 were both suppressed, showing a decrease in neuron development, while Ng2, a marker of oligodendrocytes, was elevated. These findings were buttressed by our immunocytochemistry results showing that the number of proliferating Ki67+/Tuj-1+ neuroblasts was decreased following Gro1 treatment while the number of proliferating Ki67+/Ng2+ oligodendrocyte progenitors was increased.

Treatment of HT-22 hippocampal neurons with Gro1 led to very similar results. With this cell line, we also found reduced DCX and Tuj-1 expression, as well as induction of SA-β gal, and a marked increase in the number of senescence neurons. Our results are supported by others showing that chemokine signaling via the Gro1 receptor CXCR2 in human fibroblasts reinforces senescence [60]. We further confirmed that Gro1 may limit the proliferation of progenitors of neuronal lineage via senescence by demonstrating reduced SA-β gal as well as markedly upregulated DCX expression in Gro1-suppressed murine NPC indicative of increased numbers of neuroblasts. Concordant with our previous observations, PDGFαR was downregulated in cells where Gro1 was low. Senescence is accompanied by a decrease in apoptosis [56, 59, 61], and we found that Gro1 suppressed cleaved caspase 3 expression in both murine NPC and HT-22 cells. At the same time, oligodendrocyte progenitor markers were increased, in agreement with the findings that Gro1 increases proliferation and survival of oligodendrocytes [42–44, 62]. Together, these results indicate that Gro1 induced in response to inflammation shifts hippocampal neurogenesis toward oligodendrocytes, suppressing new neuron development.

Senescent cells secrete the full array of chemokines, cytokines, and growth factors, a phenomenon termed senescence-associated secretome (SAS) [63–65]. In multiple cell types, Gro1 appears to be a part of SAS [54, 63, 66]. Some of these secretome factors may actually enhance the senescence phenotype [59, 65], as evidenced by Gro1 induction of senescence in cancer-associated fibroblasts via an autocrine loop [67]. Our data suggest that Gro1 induced in response to inflammation may trigger senescence in neuronal stem cells or neuroblasts, but not in astrocytes or oligodendrocytes. Further studies are required to unravel these cell-specific Gro1 effects.

Further, our in vivo results show that Gro1 overexpression in the hippocampus of newborn mice also results in decreased DCX expression. In the newborn brain, expression of SA-β gal in Gro1-treated mice was undetectable, while p16 was upregulated. As the marker of oligodendrocyte progenitors PDGFαR was increased, it is likely that this cell cycle arrest was specific to neuroblast proliferation.

LPS treatment evokes strong systemic inflammation in a sequence of events, including blood-brain barrier leakage [68], massive peripheral immune cells infiltration [69], and neuroinflammation. Proinflammatory cytokines are released in the periphery, and cytokines such as IL-1β, TNF-α, and IL-6 are induced in the hippocampus in microglia and astrocytes [18]. Cytokines have been linked to detrimental effects on neurogenesis [17–19, 23], and we and others [31] show that proinflammatory cytokines stimulate Gro1 expression. We demonstrate here that IL-1β is markedly induced in the hippocampus in response to inflammation. Sustained IL-1β expression also results in infiltration of neutrophils and macrophages, and the presence of immune cells is coincident with upregulation of Gro1 in the hippocampus [70]. Therefore, in the course of inflammation, Gro1 may be released from all these multiple sources [71, 72].

Following LPS treatment, we found significantly induced Gr0o1(Gro1) in the male hippocampus and a blunted response in females. This may be attributed to the blunted IL-1β response to LPS, which we observed both in female mice and in male mice treated with E2.

The hippocampus contains estrogen receptors ERα and ERβ [73, 74], and hippocampal NPC also express both receptors [75]. Estrogen has been shown to exert a dual effect on Gro1. E2 suppresses Gro1 expression in rodent models at inflammatory sites limiting LPS-induced recruitment of neutrophils, thus limiting Gro1 delivery to the hippocampus [76]. In addition, during acute inflammation, estrogens have been shown to decrease Gro1 expression by enhancing the production and limiting the degradation of the NFκB inhibitor nuclear factor of kappa light polypeptide gene enhancer in B cells inhibitor, alpha (IκBα). This arrests NFκB nuclear translocation and suppresses Gro1 transcription [76, 77].

Our results suggest that during inflammation, E2, by dampening Gro1 response, may, to some extent, protect female neurogenesis from detrimental acute intense inflammatory stimuli. Sexual dimorphism in Gro1 response to inflammation may therefore be contingent on the level of E2 in females.

A number of limitations should be considered. First, the mechanisms underlying cell-specific effects of Gro1 in NPC, with induced p16 in neuroblasts but suppressed expression in glial progenitors, remain unknown. Second, we tested the effects of LPS on hippocampal Gro1 expression in males and females but did not evaluate how the stage of the estrus cycle in females might further influence outcomes. Third, E2 pellets were implanted in males with intact levels of testosterone, a hormone that can also have protective effects on neurogenesis. Nevertheless, despite these limitations, this study broadens our knowledge and opens new approaches for understanding mechanisms underlying new neuron development in the adult brain.

## Conclusions

We present the first direct evidence that the chemokine Gro1 activates senescence in hippocampal neuronal lineage and demonstrate sex-dependent effects of systemic inflammation on Gro1 and subsequent changes in adult hippocampal neurogenesis. The selective vulnerability of hippocampal neurogenesis to inflammation further underscores the need to understand the mechanisms underlying the effects of inflammation and sex dimorphism on the development of new neurons.

## Abbreviations

bFGF: Basic fibroblast growth factor
DAPI: 4′,6-Diamidino-2-phenylindole
DCX: Doublecortin
DMEM: Dulbecco’s modified Eagle medium
ECL: Enhanced chemiluminescence
EGF: Epidermal growth factor
EGFP: Enhanced green fluorescent protein
FBS: Fetal bovine serum
FGF2: Fibroblast growth factor 2
GAPDH: Glyceraldehyde-3-phosphate dehydrogenase
GDNF: Glial cell-derived neurotrophic factor
GFAP: Glial fibrillary acidic protein
Iba1: Ionized calcium-binding adapter molecule 1
IκBα: Nuclear factor of kappa light polypeptide gene enhancer in B cells inhibitor alpha
NeuN: Neuronal nuclear antigen
NFκB: Nuclear factor kappa-light-chain-enhancer of activated B cells
Ng2: Neural glial antigen 2
Olig1,2: Oligodendrocyte transcription factor 1 and 2
PBS: Phosphate-buffered saline
PDGFαR: Platelet-derived growth factor α receptor
PSA-NCAM: Polysialylated-neural cell adhesion molecule
PVDF: Polyvinylidene difluoride
RIPA: Radioimmunoprecipitation assay
S100β: S100 calcium-binding protein B
SA-β-gal: Senescence-associated β galactosidase
SEM: Standard error of the mean
Sox2: Sex-determining region Y-box 2
TBST: Tris-buffered saline
Tuj-1: Neuron-specific class III beta-tubulin
